# Emergent function, not microbial conformity: functional redundancy and the limits of taxonomic inference in microbiome genomics

**DOI:** 10.1099/mgen.0.001831

**Published:** 2026-08-25

**Authors:** Rebecca Lewandowski

**Affiliations:** 1 Department of Molecular and Cellular Biology, University of Arizona, 1401 E University Blvd, Tucson, AZ 85721, USA

**Keywords:** functional redundancy, host–microbiome interactions, metagenomics, microbiome genomics, taxonomic inference

## Abstract

Microbiome genomics has achieved remarkable resolution of community structure, yet composition alone remains an unstable basis for inferring host-relevant biology. That instability reflects a broader interpretive problem in which taxonomically distinct communities can converge on similar outputs, while superficially similar communities can diverge in behaviour because of strain variation, gene content, regulatory state, ecological context, spatial organization and host physiology. Functional redundancy is therefore better understood not as a reserve of interchangeable organisms but as a distributed functional architecture through which host-relevant outputs can persist across variation in membership. The central question for microbial genomics is not whether composition matters, but when community structure can be expected to predict function, host consequence or recovery. A more rigorous framework must distinguish membership from encoded capacity, realized activity, ecological interaction and host-relevant effect, while also recognizing that host physiology and spatial context shape which microbial functions become possible and which outputs are ultimately encountered. Progress will depend first on matching the evidentiary layer to the claim and then on selecting proportionate additions, from strain-resolved genomics and pathway-level interpretation to targeted metatranscriptomic, metaproteomic, metabolomic, spatial, perturbation-recovery or host-response measurements. In that framework, reproducibility may reside less in recurring taxa than in conserved biological outputs, and restoration less in compositional resemblance than in recovery of the functions and host-facing consequences that were actually disrupted.

**Impact Statement**

This Perspective argues that the interpretive problem in microbiome genomics no longer surrounds simply who is there, but when sequence-resolved community structure predicts the outputs that matter to the host. It asks two central questions: when do taxonomic patterns justify functional claims, and what does restoration mean if host-relevant function can persist even when microbial composition differs? By reframing functional redundancy as distributed functional architecture, the article shows why reproducibility and restoration cannot be judged by taxonomic recurrence alone. It argues instead for a more exacting future in which microbial genomics separates membership from encoded capacity, realized activity, ecological interaction and host consequence and pairs community profiling with strain-resolved analysis, pathway-level interpretation, multi-omic integration, spatially informed sampling, perturbation-recovery design and host-response readouts. The aim is to move microbial genomics towards a more powerful interpretive framework, one in which precision lies not only in resolving communities but in explaining what their variation means for the host.

## Data Summary

No new data, code or software were generated for this article.

## Introduction

A central achievement of microbiome genomics has been to make microbial communities sequence-resolved at scale, advancing from taxonomic surveys to population- and strain-level views of genomic diversity across human cohorts [1–3]. The resulting precision, however, has encouraged an interpretive shortcut in which community membership is treated as a proxy for what the microbiome is doing. That shortcut is increasingly untenable. Taxonomic structure alone does not reliably specify metabolic activity, ecological behaviour or host consequence, and these layers can diverge substantially across communities, conditions and even strains within the same species [4–8]. The central problem for microbial genomics is therefore not only how to describe communities more precisely but also how to infer biological meaning from them more rigorously.

That interpretive habit followed from the structure of the data itself. Sequencing made taxonomic structure measurable at scale, and taxonomic structure had practical advantages as an analytic object because it was countable, portable across cohorts, statistically tractable and visually persuasive in comparative datasets. As a result, community composition became the most legible representation of the microbiome and was increasingly asked to carry explanatory weight beyond what it could securely support.

Taxonomic structure remains valuable because it can reveal major perturbations, identify candidate ecological shifts and define the starting point for deeper mechanistic work. The problem begins when composition is treated as though it were itself evidence of function, recovery or biological consequence. Healthy individuals can differ markedly in gut microbial composition, arguing against any single ideal community template [2, 3]. Superficially similar communities can still conceal important functional differences when strain content, accessory genes, transcriptional state, ecological interaction or host context diverge [5, 8, 9].

**Fig. 1.**
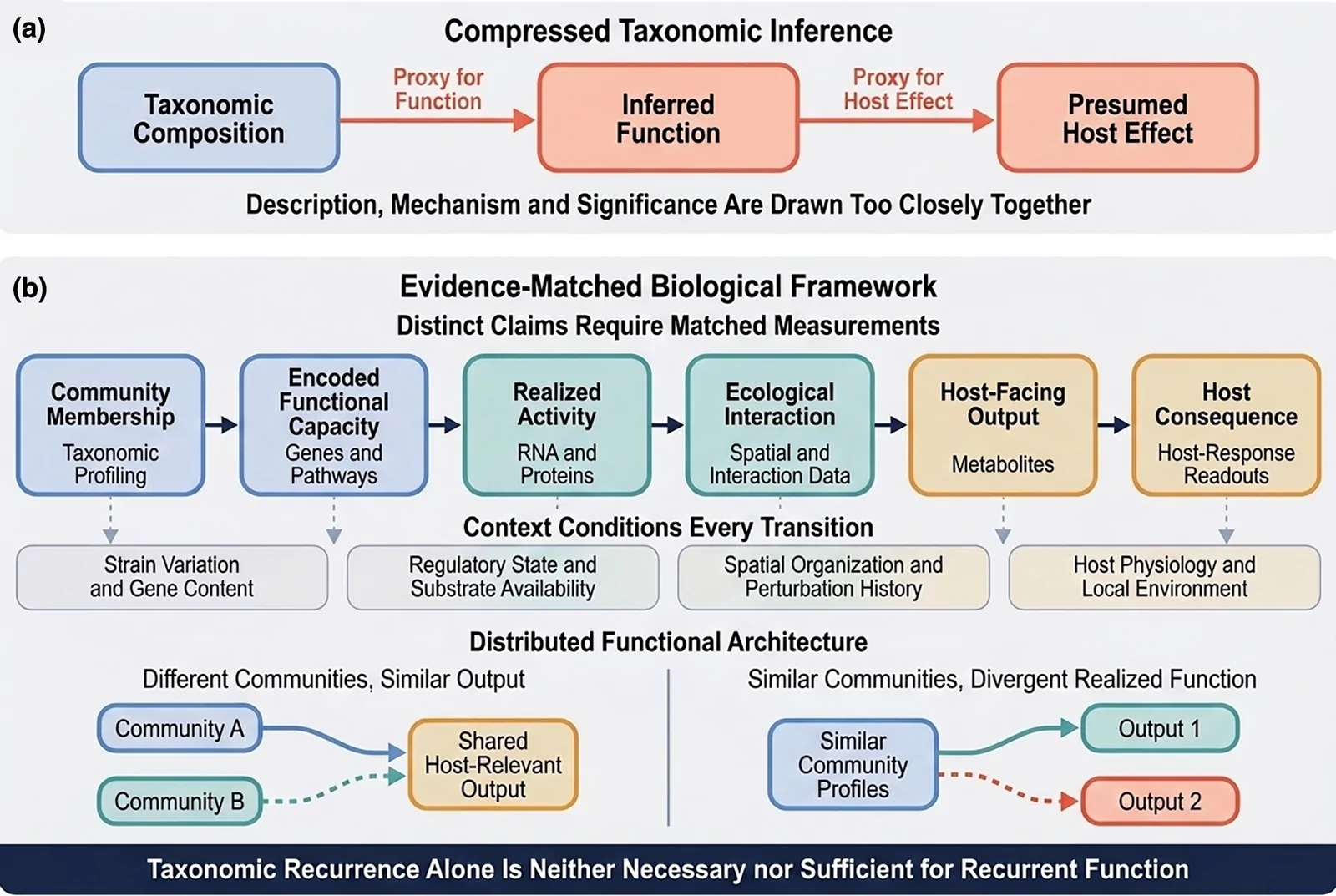
Separating taxonomic description from biological inference. (**a**) Compressed taxonomic inference treats community composition as a proxy for function and host effect, drawing description, mechanism and significance too closely together. (**b**) The evidence-matched framework separates community membership, encoded functional capacity, realized activity, ecological interaction, host-facing output and host consequence. Each layer supports a distinct class of claim; encoded capacity alone does not establish activity, metabolite production or host consequence. Relationships between layers are conditioned by strain variation and gene content, regulatory state and substrate availability, spatial organization and perturbation history and host physiology and local environment. Distributed functional architecture refers to the organization of host-relevant function across multiple organisms, genes, substrates and interactions. Taxonomically distinct communities can therefore converge on a similar output, while similar community profiles can diverge in realized function. Arrows represent conditional biological relationships rather than deterministic equivalence or demonstrated causality.

This interpretive problem presses microbial genomics towards a more exacting standard of inference. The central question is no longer simply who is there, but when sequence-resolved community structure predicts the outputs that emerge from microbial interaction, compensation and host constraint. Here, distributed functional architecture refers to the organization of a host-relevant function across multiple organisms, genes, substrates and interactions, such that the output may persist even when the same taxonomic carriers do not recur. The resulting framework replaces a compressed chain from taxonomic composition to presumed function and host effect with staged inference across community membership, encoded capacity, realized activity, ecological interaction, host-facing output and host consequence (Fig. 1).

## When taxonomy became too much of the explanation

The problem was not that taxonomy became central. The problem was that it became sufficient. Once taxonomic structure served as the dominant language of microbial comparison, it also began to serve as a proxy for function, resilience and host consequence. The result was an interpretive compression in which description, mechanism and significance were drawn too closely together.

Taxonomic signatures became attractive not only as descriptive outputs but also as explanatory ones. Community differences increasingly came to signify ecological dysfunction, while movement towards an expected community profile was readily read as biological restoration. Under that pressure, dysbiosis expanded into a term that could blur compositional difference, ecological instability and pathological significance [10]. Difference itself could then begin to function as evidence of dysfunction even when functional impairment had not been established [11].

Microbiome research still carries the residue of that measurement-driven logic. Taxa are easily compared across datasets, so taxonomic profiles continue to receive interpretive priority even when the underlying question concerns function, host consequence or resilience. Community composition can register that change has occurred, but it cannot by itself determine whether that change reflects injury, adaptation, neutrality, transience or compensation.

Microbial genomics is now well positioned to confront this limitation directly, but only with greater discipline about what taxonomic data can justify and when explanation must move instead to pathway capacity, expressed activity, metabolite production, ecological interaction or host consequence.

## Functional redundancy as system architecture

Functional redundancy is frequently reduced to the claim that different microbes can do the same thing. That captures part of the issue, but the fuller interpretation lies at the level of organization. Microbial communities can preserve host-relevant outputs through capacities distributed across multiple members, substrates and interactions. Functional continuity may therefore reside less in any single organism than in the organization of the system that links encoded capacity, ecological exchange and host context [12, 13]. Redundancy in this sense lies less in interchangeable organisms than in the ability of the community to preserve host-facing behaviour even as the organisms contributing to that behaviour, the substrates moving through the system and the interactions linking them change [12, 13].

This is easiest to see when function is assembled through cross-feeding and metabolic exchange rather than delivered by one stable taxonomic carrier. Short-chain fatty acid production is often treated as though it were a simple property of named taxa, yet the host-facing output may depend on trophic organization, substrate availability and metabolic handoff across the community. Acetate and lactate produced by one set of organisms can be converted into butyrate by another, allowing similar metabolic consequences to arise through different community arrangements under different dietary conditions [8, 14]. The relevant biological unit is therefore not always the organism itself, but the distributed route by which carbon is transformed into a host-exposed product.

Bile acid metabolism illustrates the same principle in a more explicitly host-microbial form. The host provides the initial bile acids, but the downstream products it ultimately encounters emerge from microbial deconjugation and further conversion steps that may be distributed across multiple community members rather than tied to one organism alone [15]. Similar logic applies to mucus utilization, aa metabolism and vitamin biosynthesis, where different microbial contributors can help preserve host-relevant output even as community membership changes [16–18].

Seen this way, redundancy is not a reserve bench of interchangeable organisms. It is a property of system architecture. A host-relevant function may depend on the broader arrangement of capacities across the community and on the substrates and interactions that permit those capacities to be carried out. Under those conditions, taxonomic deviation does not necessarily indicate functional loss, and taxonomic restoration does not necessarily indicate functional recovery.

This does not make redundancy universal or symmetrical. Broad outputs such as short-chain fatty acid production are often buffered because multiple taxa can contribute through overlapping fermentation pathways and cross-feeding arrangements [8]. Narrower functions, by contrast, may depend on a much smaller set of organisms carrying specialized genes, as in bile acid 7α-dehydroxylation, which is associated with a relatively restricted group of microbial contributors carrying the bile acid–inducible (*bai*) genes [15]. Functional redundancy is therefore itself structured rather than uniform across all host-relevant processes. Broad ecological or metabolic functions may be more resilient to turnover than narrow functions requiring specific enzymatic steps, specialized lineages or particular spatial conditions [12, 13]. The important distinction is not between redundancy and its absence, but between functions that are broadly substitutable and those that are not.

## When different communities yield similar outputs

If redundancy is sufficient at the level of function, compositional difference need not change what the host ultimately receives. Healthy individuals can harbour markedly different microbial compositions, which argues against any single ideal community template and against the assumption that one taxonomic arrangement is uniquely capable of supporting host health [2, 3, 7]. What may be conserved is not membership itself, but the outputs most relevant to the host.

Taxonomic change is not itself the biological event the host experiences. What the host encounters are the products and consequences of microbial activity, including metabolites, inflammatory and barrier effects, nutrient-processing outcomes and the downstream effects of ecological stability or disruption. Different community configurations may therefore produce similar host-facing biology even when they differ compositionally, provided that the relevant pathways and interactions remain available. Species identity can inform function, but it is not a sufficiently stable surrogate on its own [8]. The more informative question is therefore not simply which taxa differ, but whether the outputs most relevant to the host remain preserved, altered or redistributed [7, 14].

Vitamin biosynthesis offers one such example. Some vitamins shaped by the gut microbiome are supplied not by one fixed organism but through biosynthetic capacity distributed across members of the community, often within cooperative trophic networks. Gut microbes differ in whether they encode complete vitamin biosynthetic pathways or instead depend on salvage of intermediates and vitamers produced by neighbouring microbes or supplied by the host, so function can persist through networked metabolic exchange rather than continuity of the same taxonomic carriers alone [18]. Under those conditions, substantial taxonomic variation does not necessarily imply loss of the host-facing output if the underlying biosynthetic architecture remains intact.

For that reason, compositional difference cannot, on its own, be equated with dysfunction. If two individuals differ sharply in community structure yet maintain similar metabolite exposure, pathway activity or ecological stability, then deviation from a reference profile does not by itself establish biological impairment. Some differences may instead reflect alternate but viable configurations of a functional system. A field that overreads composition will mistake variability for pathology.

That problem carries a practical consequence for interpretation. Deviation from a reference community becomes biologically meaningful only when linked to an appropriate functional comparator, such as altered pathway capacity, expressed activity, metabolite production or host response, rather than taxonomic difference alone [5, 14]. But that shift in emphasis also changes the translational question. If host-facing function can persist across substantially different microbial configurations, then restoration cannot be inferred from compositional normalization alone [19, 20]. The question is no longer whether a community has been made to resemble a reference state, but what, exactly, has been restored.

## When similar communities yield different outputs

The reciprocal problem is equally important. Superficially similar communities can produce very different biology. A shared species label does not specify strain content, accessory genes, mobile elements, prophage state, transcriptional activity, metabolic flux, spatial organization or host environment. Similarity in community composition can therefore conceal substantial divergence in biological behaviour.

This is where the interpretive value of microbial genomics becomes increasingly clear. Closely related strains may differ in substrate use, immune interaction, ecological competitiveness, metabolite production and carriage of toxin, antimicrobial-resistance or other health-relevant genes because the relevant determinants are carried in variable gene content rather than in the species label alone. Strain-resolved profiling can therefore distinguish persistence, replacement, transmission and accessory-gene carriage that species-level abundance collapses. This improves inference about encoded capacity, but it does not establish that those genes are expressed or that their products reach the host. Strain differentiation based only on SNPs can also miss structural variation arising from insertions, deletions, recombination, horizontal gene transfer and genomic rearrangements, including sequence inversions. Synteny-based analysis shows that these modes of diversification vary among microbial species and can distinguish strains that appear similar under SNP-centred comparisons [21]. Accessory genomes, prophages and other mobile elements can further reshape behaviour without necessarily producing an obvious shift in species-level profiles [5, 9, 22]. The same named organism can therefore imply different biological possibilities in different communities.

One concrete example comes from *Bacteroides vulgatus*, where lysogenic infection by phage BV01 broadly alters the bacterial transcriptome and disrupts expression of *tspO*, a regulator linked to bile acid metabolism. In the lysogen, this is associated with reduced bile salt hydrolase activity and diminished bile acid deconjugation, showing how the same named bacterium can carry different functional implications depending on its mobile genetic context [22]. Species identity alone would not resolve that difference.

But the inferential instability runs deeper than species labels. Sequence-based functional profiling, whether based on annotated genes or reconstructed pathways, estimates encoded capacity. It establishes that a function may be possible, but not that the relevant genes are expressed, enzymes are active, substrates are available, flux occurs or the resulting product reaches the host. Metatranscriptomics narrows that gap by measuring microbial RNA under the sampled condition, while metaproteomics adds evidence that microbial proteins are present. Metabolomics moves closer to chemical output, but the observed metabolite pool is a net state shaped by microbial and host production, consumption, transport, diet and clearance. These layers add proximity to realized biology, not automatic mechanism. RNA abundance does not establish protein activity or metabolic flux, and metabolite abundance does not identify producer, pathway, direction of flux or biological effect on its own. Their value lies in testing whether encoded potential is accompanied by expression, product formation and host response [5, 6, 14, 23].

Whether encoded capacity becomes realized activity remains contingent on ecological and host context. A pathway may be present in a metagenome yet remain quiescent under one set of ecological conditions and become dominant under another. Diet, inflammation, oxygen gradients, substrate competition, host signalling and spatial location within the gut all help determine which genomic capacities are actually used. Community composition flattens those contingencies, and functional annotation may do so as well when it is not linked to expression, metabolite production or host response [5, 14].

This problem is particularly visible when local biology is compressed into bulk community descriptions. Communities that appear similar in stool may still differ in mucosal proximity, regional localization or engagement with host tissue. Host-relevant activity that is inducible, spatially restricted or enriched at the mucosal interface may therefore be only partially represented in bulk faecal sampling [20, 24].

This two-sided instability matters directly for reproducibility. If different communities can support similar biology and similar communities can support different biology, then taxonomic recurrence alone cannot bear the full burden of replication. Some failures to reproduce taxonomic signatures may reflect genuine biological heterogeneity. Others may arise because taxonomic patterns are being asked to stand in for biological layers they do not stably capture. Under those conditions, recurrent compositional signatures will be a poor proxy for recurrent function, recurrent host consequence or recurrent mechanism [5, 14].

## The host as part of the genomic problem

Microbial communities do not operate independently of the host. From the standpoint of host consequence, what matters is not always which organism generated a signal but whether the system still produces, modulates or buffers the outputs the host actually encounters. Host physiology therefore helps determine which microbial capacities become possible, amplified, constrained or irrelevant [9, 15, 24]. Mucus composition, epithelial signalling, immune filtering, nutrient flow, transit, oxygen gradients, bile acid chemistry, circadian timing and host genetics all help define the environment in which microbial behaviour occurs [9, 15, 24].

Host constraint helps explain why encoded microbial capacity does not automatically translate into host-relevant effect. A gene catalogue does not act in isolation, but within a host-defined chemical and spatial landscape. Host-derived bile acids, for example, do not merely provide substrates for microbial modification. They also create a chemical environment that some microbes tolerate and transform more effectively than others, so the bile acid pool ultimately returned to the host reflects both host-defined constraint and selective microbial processing rather than microbial capacity alone [15]. The host is therefore not simply downstream of microbial function. It helps organize the conditions under which microbial function becomes possible.

Host genetic regulation reinforces the same point from another angle. Host genotype can shape the intestinal conditions under which microbes persist and interact, so community organization is not merely observed but partly constrained by host biology itself [9]. In the clearest cases, host glycan features are associated with enrichment of microbial genetic capacity to use those substrates, as when an N-acetylgalactosamine (GalNAc) utilization gene cluster in *Faecalibacterium prausnitzii* is more common in individuals who secrete a type A oligosaccharide terminating in *N*-acetylgalactosamine [9]. The same microbial capacity may therefore not have the same probability of persistence, expression or host relevance across hosts. What appears compositionally similar can be filtered into different functional outcomes because the host is shaping the field of possible interactions.

Spatial context adds a further layer of instability to taxonomic inference. Stool remains indispensable for longitudinal and population-scale work, but it is a distal downstream readout rather than a direct record of where local microbial activity occurred. Regional variation, mucosal proximity, patchy ecological niches and local host interactions can shape function in ways that bulk faecal profiles flatten, attenuate or fail to preserve, especially once microbial distribution is recognized as anatomically patterned rather than spatially interchangeable along the gut [24]. A community may therefore appear stable in stool even while host-relevant microbial activity is being reorganized locally at the epithelial interface or within specific intestinal regions.

A future-facing microbial genomics must therefore treat the host not as background noise but as part of the causal architecture. Functional adequacy may depend less on recreating a specific microbial census than on whether the outputs most consequential to the host are preserved, restored or maladaptively altered under current conditions [9, 15, 24].

## Reproducibility beyond recurring taxa

The same logic that unsettles taxonomic inference also unsettles how reproducibility is defined. Once community composition is no longer treated as a self-sufficient surrogate for function, reproducibility cannot be reduced to recurrence of taxa alone. In at least some contexts, the more reproducible signal may lie in pathway-level, metabolic, ecological or host-response patterns rather than in repeated taxonomic membership [14]. Multi-omics studies in inflammatory bowel disease illustrate this shift because host-relevant states emerge more clearly when genomic, transcriptomic and metabolic layers are examined together than when disease is inferred from membership alone [5].

That observation should not weaken standards for study design. It should refine them. Within microbial genomic interpretation, the relevant question is not only what layer of biology has been measured but also what kind of claim that layer can support. Membership, encoded capacity, realized activity, ecological interaction and host consequence are not interchangeable, and none should be allowed to stand in uncritically for the others.

These recommendations should be treated as a hierarchy rather than an all-or-none requirement. The first priority is the lowest-cost, highest-yield correction: define the biological claim before selecting the evidentiary layer. Where shotgun metagenomes are already available, strain-resolved profiling, gene-level analysis and pathway reconstruction can deepen inference without new specimen collection, although all remain measures of encoded capacity rather than realized function [5, 9, 21, 22]. At the same tier, studies should prespecify the limited set of covariates most likely to alter the measured layer or exposure rather than attempt an exhaustive inventory of every possible confounder. For gut studies, recent antibiotics and other microbiome-active medications, diet or substrate exposure, sampling timing, stool form or transit and analytical batch will often deserve priority.

A second tier adds one orthogonal functional measurement selected to match the claim. Metatranscriptomics is most useful when the question concerns microbial expression, targeted metabolomics when it concerns a defined chemical output, metaproteomics when the presence of microbial proteins is central and focused host-response assays when the claim concerns epithelial, immune or physiological consequence. A targeted assay with a clear interpretive role will often provide more value than broad multi-omic accumulation without a defined evidentiary purpose [5, 6, 14, 23].

The most resource-intensive designs should be reserved for questions that require them. Spatially resolved or intestine-proximal sampling is justified when location or host-interface engagement is central, while dense longitudinal and perturbation-recovery designs are justified when the claim concerns resistance, fragility or recovery rather than static difference. Nested designs, in which these measurements are obtained from a mechanistically selected subset, can preserve interpretive value when sample volume, funding or logistics preclude their use across the full cohort [19, 20, 24, 25].

Artificial intelligence may eventually help connect these evidence layers by identifying nonlinear relationships across genomic, transcriptomic, metabolic, spatial and host-response data. Mechanistically constrained graph-learning approaches that encode pathways and cross-feeding rather than relying on taxonomic correlations alone illustrate one direction for this work [26]. Their value, however, will remain bounded by the measurements, sampling design and biological assumptions supplied to the model. Computational integration can organize cross-layer evidence and generate testable predictions, but it cannot convert encoded potential into demonstrated activity, assign metabolite provenance or establish host consequence when those quantities were not measured.

The translational consequence is difficult to avoid. If reproducibility shifts towards pathways, metabolites, ecological responses and host-integrated outputs, then microbiome intervention cannot be judged by taxonomic movement alone. Personalized colonization resistance and post-antibiotic recovery studies already suggest that adding named organisms does not straightforwardly restore host-relevant biology and, in some contexts, may delay reconstitution of the indigenous system [19, 20]. Restoration therefore cannot be defined as movement towards a preferred compositional profile. If host-relevant function can persist across distinct microbial arrangements, restoration must instead be judged by recovery of the outputs, interactions and host consequences that were actually lost or maladaptively altered [5, 19, 20].

This is not only a methodological correction but also a change in what microbiome precision should mean. Commercial microbiome testing, consumer probiotic narratives and some clinical interpretations still often treat taxonomic deviation as though it mapped cleanly onto dysfunction and compositional resemblance as though it marked recovery. Microbial genomics is now in a position to demand a more defensible standard, one in which precision lies not in naming a supposedly correct microbial roster, but in identifying which differences alter function, ecological behaviour and host consequence in ways that endure.
